# Supplementary material for: A Likelihood Approach for Real-Time Calibration of Stochastic Compartmental Epidemic Models
Source: PLoS Comput Biol. 2017 Jan 17;13(1):e1005257. doi: 10.1371/journal.pcbi.1005257 (PMC5240920; doi:10.1371/journal.pcbi.1005257)
Supplement: S1 File — (TAR.GZ) [file pcbi.1005257.s014.tar.gz › HSPH_Online-SI-Revision/IPoi09/n100-mild/n100-mild_BENCH_table-8.pdf]

1	R0	{1.24125, 3.38982}	{1.19264, 2.01465}	{{1.19264, 2.01465}, {1.19264, 2.01465}}	{3.91621, 40.5676}	inf	218.	1010.92	{299., 1616.}	380.008	0.717625	newly	544.	3650.5	{1539., 5242.}	587.469	0.904923	Reff	1.2258	1.07943	{0.983833, 1.16632}	11.9405	ar	34926.	28089.8	{28394., 32509.}	19.5733
2	R0	{1.29554, 4.51878}	{1.30094, 3.11158}	{{1.30094, 3.11158}, {1.30094, 3.11158}}	{0.416815, 31.1411}	inf	496.	1390.82	{119., 2953.}	199.881	0.467135	newly	1039.	3588.47	{326., 7065.}	263.891	0.555833	Reff	1.26727	1.20035	{1.06993, 1.29352}	5.8083	ar	42828.	42208.9	{41355., 43623.}	2.1929
3	R0	{1.2443, 3.64292}	{1.37363, 2.81014}	{{1.37363, 2.81014}, {1.37363, 2.81014}}	{10.3938, 22.8602}	inf	288.	1741.14	{431., 3162.}	513.526	0.742308	newly	581.	5469.45	{1607., 9356.}	850.07	0.932889	Reff	1.22536	1.26498	{1.16355, 1.34984}	6.80733	ar	37087.	48965.7	{48188., 49769.}	32.0294
4	R0	{1.38465, 3.43331}	{2.39007, 5.7571}	{{2.15896, 5.03398}, {2.68593, 6.66951}}	{72.6117, 72.4394}	inf	73.	290.836	{42., 607.}	314.189	0.533037	newly	229.	884.269	{135., 2039.}	300.143	0.522677	Reff	1.38185	2.39756	{2.14881, 2.68324}	73.5033	ar	49511.	86652.6	{83352., 91668.}	75.0572
5	R0	{1.25409, 4.1068}	{1.47527, 3.82941}	{{1.47608, 3.83841}, {1.47608, 3.83841}}	{17.637, 6.75447}	inf	293.	1477.42	{214., 2944.}	414.4	0.638944	newly	618.	3781.77	{603., 7021.}	516.965	0.703813	Reff	1.23932	1.40229	{1.33339, 1.46466}	13.1506	ar	38571.	56677.5	{55996., 57609.}	46.9434
6	R0	{1.28821, 4.2016}	{1.24751, 2.5364}	{{1.24751, 2.5364}, {1.24751, 2.5364}}	{3.15942, 39.6325}	inf	415.	1253.93	{356., 2163.}	208.482	0.444819	newly	847.	3935.8	{1340., 5978.}	364.675	0.618508	Reff	1.26157	1.14391	{1.02832, 1.21993}	9.32612	ar	41468.	36978.9	{35665., 38611.}	10.8255
7	R0	{1.24369, 2.56007}	{1.32501, 2.23443}	{{1.27121, 1.91565}, {1.4031, 2.55684}}	{6.5385, 13.8924}	inf	56.	239.692	{28., 724.}	350.962	0.535423	newly	202.	1252.85	{152., 3637.}	534.12	0.637497	Reff	1.24058	1.32132	{1.2599, 1.40212}	6.55874	ar	38603.	45384.2	{39465., 51856.}	17.6738
8	R0	{1.232, 3.53942}	{1.31535, 3.47086}	{{1.31535, 3.47086}, {1.31535, 3.47086}}	{6.76542, 1.93704}	inf	337.	896.167	{59., 2252.}	200.511	0.476911	newly	787.	2196.28	{171., 5102.}	208.986	0.478597	Reff	1.2117	1.25642	{1.15192, 1.3109}	5.34802	ar	34555.	44107.9	{42394., 45509.}	27.6455
9	R0	{1.36262, 3.85631}	{1.68341, 4.48092}	{{1.56331, 3.72349}, {1.90341, 5.70177}}	{23.5423, 17.7125}	inf	149.	482.163	{48., 980.}	249.914	0.51741	newly	418.	1313.35	{125., 2747.}	243.429	0.529589	Reff	1.35645	1.68661	{1.54955, 1.88036}	24.3401	ar	48410.	68111.7	{62304., 76545.}	40.6975
10	R0	{1.28702, 4.67968}	{1.19264, 2.01465}	{{1.19264, 2.01465}, {1.19264, 2.01465}}	{7.33322, 56.949}	inf	588.	963.091	{571., 1368.}	67.8417	0.209328	newly	1085.	3074.18	{1524., 4742.}	183.335	0.412635	Reff	1.24853	0.933479	{0.857317, 1.06009}	25.2334	ar	41092.	30623.2	{28697., 32509.}	25.4765
11	R0	{1.35752, 3.47129}	{1.22163, 2.29438}	{{1.19264, 2.01465}, {1.24751, 2.5364}}	{10.0098, 33.904}	inf	93.	436.316	{6., 1061.}	405.829	0.725416	newly	286.	1615.63	{15., 4436.}	500.865	0.807191	Reff	1.35065	1.20424	{1.11955, 1.28196}	10.8397	ar	44669.	31288.8	{223., 41426.}	29.954
12	R0	{1.35929, 5.83647}	{1.31535, 3.47086}	{{1.31535, 3.47086}, {1.31535, 3.47086}}	{3.23257, 40.5315}	inf	673.	1321.61	{111., 2936.}	133.408	0.416578	newly	1048.	3130.5	{295., 6249.}	221.157	0.490777	Reff	1.3216	1.22193	{1.07581, 1.30797}	7.54111	ar	46715.	44107.9	{42394., 45509.}	5.58089
13	R0	{1.28019, 3.29908}	{1.32445, 3.0465}	{{1.19264, 2.01465}, {1.47488, 4.06661}}	{6.27698, 15.4618}	inf	93.	396.44	{76., 842.}	337.634	0.579565	newly	305.	1216.88	{267., 2221.}	310.439	0.552653	Reff	1.27548	1.33691	{1.17766, 1.46302}	7.25033	ar	40941.	46760.9	{31405., 57633.}	21.6834
14	R0	{1.36661, 3.48318}	{1.32284, 2.25413}	{{1.27121, 1.91565}, {1.4031, 2.55684}}	{4.05744, 35.2853}	inf	101.	226.1	{6., 612.}	187.954	0.625549	newly	296.	1150.13	{45., 3345.}	335.676	0.700887	Reff	1.36217	1.3155	{1.26445, 1.39778}	4.32294	ar	48672.	42138.5	{40171., 51282.}	15.7149
15	R0	{1.35865, 3.84123}	{2.07057, 9.32415}	{{1.50982, 4.79544}, {2.66419, 14.2056}}	{52.6541, 143.878}	inf	112.	544.564	{35., 1348.}	408.458	0.667022	newly	273.	653.855	{48., 1759.}	178.482	0.495044	Reff	1.35217	2.08473	{1.50944, 2.5814}	54.1769	ar	48598.	78266.2	{59605., 90632.}	64.6828
16	R0	{1.22879, 3.98329}	{1.26656, 3.29313}	{{1.26656, 3.29313}, {1.26656, 3.29313}}	{3.07376, 17.3264}	inf	457.	1078.89	{103., 1999.}	166.74	0.458563	newly	913.	2637.79	{291., 4649.}	210.313	0.48307	Reff	1.20087	1.18561	{1.12263, 1.25647}	4.03123	ar	34405.	38775.1	{36839., 40290.}	12.7018
17	R0	{1.34925, 3.64524}	{1.27121, 1.91565}	{{1.27121, 1.91565}, {1.27121, 1.91565}}	{5.78395, 47.4479}	inf	135.	404.826	{43., 1203.}	236.844	0.604598	newly	291.	2207.52	{207., 5968.}	677.723	0.885418	Reff	1.34265	1.24588	{1.19672, 1.26845}	7.20739	ar	46871.	37950.	{38243., 41018.}	19.0332
18	R0	{1.33054, 2.25275}	{2.17374, 2.53119}	{{1.50856, 1.41773}, {2.7569, 3.55157}}	{63.3727, 28.0283}	inf	11.	28.52	{0., 89.}	253.091	1.16133	newly	52.	595.39	{0., 1689.}	1132.67	1.74695	Reff	1.33009	2.17256	{1.50844, 2.75684}	63.3396	ar	44336.	47075.	{1., 91353.}	92.1736
19	R0	{1.28394, 3.69859}	{1.31535, 3.47086}	{{1.31535, 3.47086}, {1.31535, 3.47086}}	{2.44638, 6.15721}	inf	236.	896.167	{59., 2252.}	304.779	0.544542	newly	569.	2196.28	{171., 5102.}	308.192	0.549618	Reff	1.26314	1.25642	{1.15192, 1.3109}	3.53163	ar	39558.	44107.9	{42394., 45509.}	11.5018
20	R0	{1.2883, 2.26493}	{2.13986, 6.67946}	{{1.30094, 2.60601}, {2.85474, 13.1646}}	{67.5876, 196.228}	inf	9.	5.47	{0., 15.}	84.7778	0.999928	newly	36.	26.35	{0., 70.}	113.917	1.49664	Reff	1.28811	2.13966	{1.30091, 2.85226}	67.5956	ar	40768.	43946.	{1., 92455.}	101.941
21	R0	{1.34098, 2.78621}	{2.30833, 3.37582}	{{1.67287, 2.12856}, {2.90743, 4.62276}}	{72.3648, 33.3681}	inf	10.	14.77	{0., 37.}	146.9	1.04321	newly	66.	185.41	{0., 454.}	278.197	1.46527	Reff	1.34068	2.30772	{1.67282, 2.90737}	72.3585	ar	44605.	50692.	{1., 92894.}	93.9315
22	R0	{1.33783, 5.95911}	{1.36609, 4.01271}	{{1.31535, 3.47086}, {1.38889, 4.25615}}	{3.15438, 32.6626}	inf	837.	1795.32	{442., 3609.}	141.62	0.421901	newly	1123.	3551.88	{915., 6657.}	233.033	0.517405	Reff	1.29617	1.25289	{1.07922, 1.36515}	5.97667	ar	45418.	48735.2	{43317., 51023.}	9.13074
23	R0	{1.33359, 3.3069}	{1.37332, 3.32493}	{{1.36035, 3.22945}, {1.47488, 4.06661}}	{3.0141, 4.68221}	inf	97.	564.375	{86., 1279.}	495.232	0.635945	newly	260.	1628.04	{316., 3916.}	536.522	0.666164	Reff	1.32804	1.35842	{1.30595, 1.45029}	2.94951	ar	46245.	49813.7	{47127., 56433.}	7.78201
24	R0	{1.30588, 2.56123}	{2.39642, 5.90891}	{{1.81179, 4.06746}, {2.87797, 7.22215}}	{83.5633, 131.469}	inf	22.	43.9859	{3., 114.}	148.592	0.501825	newly	124.	136.239	{7., 356.}	89.2435	0.588255	Reff	1.30531	2.41811	{1.9119, 2.94373}	85.3267	ar	42383.	78166.1	{44428., 93555.}	104.14
25	R0	{1.29813, 4.51849}	{1.26656, 3.29313}	{{1.26656, 3.29313}, {1.26656, 3.29313}}	{2.43196, 27.1188}	inf	361.	1078.89	{103., 1999.}	223.794	0.508143	newly	633.	2637.79	{291., 4649.}	333.259	0.590841	Reff	1.27199	1.18561	{1.12263, 1.25647}	6.79055	ar	43371.	38775.1	{36839., 40290.}	10.5968
26	R0	{1.33231, 3.99953}	{1.19264, 2.01465}	{{1.19264, 2.01465}, {1.19264, 2.01465}}	{10.4833, 49.6278}	inf	222.	1010.92	{299., 1616.}	371.659	0.711044	newly	479.	3650.5	{1539., 5242.}	678.497	0.950976	Reff	1.3147	1.07943	{0.983833, 1.16632}	17.8952	ar	44733.	28089.8	{28394., 32509.}	37.2056
27	R0	{1.30555, 4.61445}	{2.08887, 12.6634}	{{1.77826, 9.06807}, {2.53519, 17.5902}}	{59.9992, 174.43}	inf	301.	1460.03	{262., 3399.}	394.701	0.596651	newly	599.	1086.34	{240., 2480.}	118.208	0.372317	Reff	1.2885	2.07157	{1.69907, 2.51765}	60.7741	ar	42624.	81563.4	{72128., 89696.}	91.3557
28	R0	{1.26819, 5.34969}	{1.19264, 2.01465}	{{1.19264, 2.01465}, {1.19264, 2.01465}}	{5.95731, 62.3408}	inf	559.	1057.25	{566., 1526.}	105.799	0.575034	newly	884.	3602.83	{2578., 5053.}	324.227	0.910293	Reff	1.2407	1.03534	{0.932633, 1.14478}	16.5517	ar	38946.	28089.8	{28394., 32509.}	27.8749
29	R0	{1.19925, 3.00394}	{1.31023, 3.04996}	{{1.31023, 3.04996}, {1.31023, 3.04996}}	{9.25412, 1.53199}	inf	185.	579.619	{77., 1268.}	241.828	0.479801	newly	516.	1796.86	{309., 3812.}	270.949	0.496636	Reff	1.18521	1.27517	{1.2277, 1.30533}	7.59062	ar	31727.	43460.7	{42298., 44573.}	36.9832
30	R0	{1.35749, 3.75483}	{1.21514, 2.22857}	{{1.19264, 2.01465}, {1.24751, 2.5364}}	{10.4865, 40.648}	inf	91.	255.125	{6., 580.}	228.159	0.552821	newly	259.	1023.81	{28., 2776.}	328.161	0.589406	Reff	1.35287	1.20583	{1.15895, 1.24566}	10.8691	ar	48244.	31876.6	{28394., 37468.}	33.9264
31	R0	{1.27131, 4.2161}	{1.31535, 3.47086}	{{1.31535, 3.47086}, {1.31535, 3.47086}}	{3.46414, 17.6761}	inf	214.	570.667	{20., 1604.}	204.413	0.482913	newly	451.	1463.94	{78., 3899.}	254.878	0.513943	Reff	1.25808	1.27996	{1.21173, 1.31244}	2.96004	ar	38299.	44107.9	{42394., 45509.}	15.1672
32	R0	{1.31827, 6.18119}	{1.19264, 2.01465}	{{1.19264, 2.01465}, {1.19264, 2.01465}}	{9.52991, 67.4068}	inf	869.	1105.18	{817., 1480.}	30.2542	0.107954	newly	1107.	3621.91	{2458., 4886.}	227.182	0.502415	Reff	1.27462	0.974981	{0.891498, 1.10653}	23.5082	ar	45262.	30623.2	{28697., 32509.}	32.3424
33	R0	{1.20398, 1.35167}	{2.1984, 2.80624}	{{1.50856, 1.47098}, {2.77593, 3.75813}}	{82.5946, 107.805}	inf	3.	22.7	{0., 79.}	734.	1.10791	newly	20.	374.11	{0., 1333.}	1849.35	1.62285	Reff	1.20375	2.19748	{1.50854, 2.7759}	82.5531	ar	33329.	50986.3	{1., 91923.}	128.976
34	R0	{1.32776, 3.146}	{1.61306, 4.34605}	{{1.37363, 2.81014}, {2.05986, 6.93093}}	{21.4877, 49.4616}	inf	86.	300.841	{42., 607.}	275.819	0.514233	newly	292.	794.977	{84., 1709.}	214.345	0.478706	Reff	1.32356	1.81026	{1.35384, 2.7902}	36.9633	ar	44872.	66674.7	{48610., 92550.}	53.1293
35	R0	{1.279, 1.11336}	{2.4644, 2.60018}	{{1.90958, 1.84149}, {2.95165, 3.22628}}	{92.6815, 133.544}	inf	8.	66.29	{0., 153.}	795.625	1.25152	newly	51.	2084.9	{0., 4920.}	4052.04	1.95533	Reff	1.27844	2.46164	{1.90874, 2.94297}	92.5504	ar	41033.	59458.1	{1., 93393.}	108.901
36	R0	{1.20029, 1.94717}	{2.26057, 4.40852}	{{1.30222, 2.23442}, {2.95668, 6.34697}}	{88.7535, 127.107}	inf	9.	12.41	{0., 34.}	145.889	1.06961	newly	29.	117.48	{0., 254.}	412.621	1.44738	Reff	1.20009	2.26009	{1.30219, 2.95665}	88.7442	ar	33234.	46879.3	{1., 92895.}	131.053
37	R0	{1.33216, 3.69685}	{1.19264, 2.01465}	{{1.19264, 2.01465}, {1.19264, 2.01465}}	{10.4732, 45.5036}	inf	191.	786.417	{159., 1396.}	333.639	0.65381	newly	501.	3150.92	{768., 5400.}	545.126	0.809321	Reff	1.3191	1.11969	{1.04512, 1.1794}	15.1177	ar	45235.	28089.8	{28394., 32509.}	37.9024
38	R0	{1.25434, 2.04314}	{2.2813, 2.58246}	{{1.50856, 1.41773}, {2.88292, 3.55157}}	{81.8723, 37.32}	inf	23.	34.02	{0., 95.}	145.13	1.24275	newly	145.	777.5	{0., 2098.}	528.359	1.84638	Reff	1.25374	2.27989	{1.50854, 2.88289}	81.8478	ar	38662.	47326.6	{1., 92308.}	110.408
39	R0	{1.27466, 3.0741}	{1.51249, 2.74776}	{{1.43965, 2.60601}, {1.58672, 3.05492}}	{18.6581, 10.6159}	inf	125.	496.028	{78., 843.}	310.733	0.514769	newly	445.	2332.86	{504., 4152.}	433.14	0.623265	Reff	1.2688	1.49424	{1.41289, 1.57804}	17.7682	ar	40181.	59211.2	{54014., 63234.}	47.3612
40	R0	{1.33045, 2.68154}	{1.32072, 1.39473}	{{1.30222, 1.35944}, {1.36566, 1.47098}}	{2.42471, 47.9878}	inf	25.	72.5357	{0., 179.}	244.143	0.850601	newly	85.	847.821	{0., 1827.}	934.58	1.26221	Reff	1.32907	1.32069	{1.28672, 1.36425}	2.57692	ar	45951.	36808.9	{16., 48693.}	23.2795
41	R0	{1.37003, 2.83984}	{2.23211, 3.49603}	{{1.4023, 2.07266}, {2.85474, 4.62276}}	{64.3312, 35.8427}	inf	9.	14.19	{0., 37.}	145.222	0.92421	newly	52.	136.32	{0., 367.}	251.462	1.30218	Reff	1.36976	2.23153	{1.40163, 2.85286}	64.3217	ar	48926.	51284.2	{1., 92485.}	83.509
42	R0	{1.3003, 2.94885}	{1.79664, 4.98568}	{{1.4804, 3.12918}, {2.40735, 8.12091}}	{38.2609, 70.85}	inf	51.	171.529	{26., 317.}	260.4	0.506768	newly	214.	517.216	{60., 1279.}	178.835	0.426738	Reff	1.29797	1.85416	{1.4709, 2.41627}	43.1445	ar	44237.	70665.5	{56580., 88203.}	60.6346
43	R0	{1.33756, 2.92653}	{2.16944, 2.62428}	{{1.50856, 1.41773}, {2.7569, 3.68909}}	{62.3006, 21.2205}	inf	19.	23.86	{0., 80.}	137.474	1.31797	newly	61.	435.33	{0., 1529.}	712.279	1.84491	Reff	1.33705	2.16848	{1.50841, 2.75687}	62.2896	ar	47342.	42685.2	{1., 91171.}	88.1587
44	R0	{1.35801, 2.92276}	{1.96275, 4.27967}	{{1.4023, 2.23442}, {2.67853, 6.23525}}	{45.3172, 57.9173}	inf	11.	38.1964	{0., 105.}	287.175	0.681335	newly	66.	139.768	{0., 365.}	164.908	0.679599	Reff	1.35749	2.12789	{1.40156, 2.72121}	57.2183	ar	47940.	68839.5	{19., 91965.}	72.1543
45	R0	{1.31342, 4.02649}	{1.37363, 2.81014}	{{1.37363, 2.81014}, {1.37363, 2.81014}}	{4.58422, 30.2087}	inf	222.	959.455	{175., 1958.}	347.052	0.608452	newly	477.	3317.55	{754., 6216.}	604.46	0.78033	Reff	1.30142	1.32128	{1.26254, 1.36515}	3.46708	ar	42995.	48965.7	{48188., 49769.}	13.887
46	R0	{1.37919, 3.75889}	{1.44908, 3.4832}	{{1.43579, 3.38828}, {1.47608, 3.83841}}	{5.15457, 11.4179}	inf	78.	316.971	{18., 770.}	325.934	0.608227	newly	193.	995.943	{56., 2501.}	435.722	0.704358	Reff	1.37534	1.44702	{1.401, 1.44079}	5.24998	ar	49157.	53296.1	{52801., 56062.}	14.182
47	R0	{1.37458, 3.56117}	{2.27654, 9.08367}	{{1.74592, 5.58477}, {2.87181, 13.004}}	{65.617, 155.27}	inf	90.	222.65	{35., 457.}	170.722	0.391515	newly	236.	317.65	{42., 842.}	86.8997	0.338617	Reff	1.37118	2.2865	{1.73654, 2.86408}	66.754	ar	48478.	83747.5	{70877., 93141.}	72.7537
48	R0	{1.33393, 3.78296}	{1.21922, 2.99437}	{{1.22544, 3.07157}, {1.22544, 3.07157}}	{8.59969, 20.8458}	inf	172.	529.643	{89., 1237.}	227.949	0.468123	newly	441.	1428.29	{234., 3114.}	242.339	0.47696	Reff	1.3221	1.18572	{1.13467, 1.21801}	10.3154	ar	44885.	34402.7	{33368., 36227.}	23.3536
49	R0	{1.24918, 2.39046}	{1.43965, 2.60601}	{{1.43965, 2.60601}, {1.43965, 2.60601}}	{15.2476, 9.01709}	inf	41.	430.56	{32., 839.}	961.463	0.891526	newly	241.	2025.2	{165., 3948.}	753.577	0.815636	Reff	1.2448	1.41936	{1.4012, 1.43717}	14.0238	ar	35969.	54202.8	{53240., 55085.}	50.6932
50	R0	{1.28281, 2.3032}	{1.40791, 2.0303}	{{1.39162, 1.89883}, {1.4023, 2.07266}}	{9.75226, 13.962}	inf	28.	242.257	{25., 470.}	770.306	0.756434	newly	129.	1683.23	{222., 3346.}	1205.54	0.941985	Reff	1.28046	1.40433	{1.37285, 1.40077}	9.67379	ar	40223.	52239.9	{50274., 52355.}	29.8757
